# Confined placental mosaicism of trisomy 6 detected through genome‐wide NIPT was associated with placental abruption

**DOI:** 10.1002/ccr3.5155

**Published:** 2021-12-05

**Authors:** Miyuki Nishiyama, Seiji Wada, Fuyuki Hasegawa, Yohji Uehara, Mamoru Ozaki, Kenichiro Hata, Yushi Ito, Haruhiko Sago

**Affiliations:** ^1^ Center for Maternal‐Fetal, Neonatal and Reproductive Medicine National Center for Child Health and Development Tokyo Japan; ^2^ National Research Institute for Child Health and Development Department of Biobank Tokyo Japan; ^3^ Division of Genomic Medicine, Department of Advanced Medicine Medical Research Institute, Kanazawa Medical University Ishikawa Japan; ^4^ Department of Maternal‐Fetal Biology National Research Institute for Child Health and Development Tokyo Japan

**Keywords:** cell‐free DNA, confined placental mosaicism, noninvasive prenatal testing, placental abruption, trisomy 6

## Abstract

Confined placental mosaicism (CPM) leads to discordant noninvasive prenatal testing (NIPT) results. We describe a very rare case of CPM of trisomy 6 detected through genome‐wide NIPT. This case was associated with placental abruption, which might suggest an association between certain types of CPM detected by NIPT and pregnancy complications.

## Graphical abstract

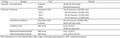

## 1 INTRODUCTION

Confined placental mosaicism (CPM) is defined as a chromosomally abnormal cell line restricted to the placenta, while the fetus is chromosomally normal. Confined placental mosaicism can be identified by chorionic villus sampling (CVS) for a cytogenetic prenatal diagnosis with discrepancies involving mosaicism in the chorionic villi. When a rare autosomal trisomy (RAT, defined as any autosomal trisomy other than trisomy 21, trisomy 18, or trisomy 13) is detected in CVS; CPM accounts for 97% of these cases.^1^ Confined placental mosaicism pregnancies have been reported to be associated with spontaneous miscarriage, intrauterine growth restriction, intrauterine fetal death, preterm birth, and stillbirth; however, most result in uneventful term pregnancies.^2^ The possible association between CPM and adverse pregnancy outcomes remains unclear and the perinatal outcomes of CPM—especially in RAT—have not been well investigated.

With the arrival of noninvasive prenatal testing (NIPT) using cell‐free DNA (cfDNA), CPM can be prenatally detected through NIPT. In comparison with CVS, NIPT is more sensitive in the detection of CPM that is restricted to a small part of the placenta, since the fetal fraction of cfDNA is derived from the villi.^3^ Although NIPT is performed with a focus on common aneuploidies,^4^ it has recently become possible to perform a genome‐wide (GW) analysis, which could reveal the presence of RATs. In recent data from 10 reports on GW‐NIPT of 196,662 samples, the weighted average rate of positive results for RATs was 0.32%, and trisomy 7, 15, 16, and 22 were the most commonly represented abnormalities in RATs (≥10% of RATs each).^5^ Trisomy 6 is rarely observed in GW‐NIPT, CVS, and products of conception^5^; therefore, trisomy 6 CPM is extremely rare. We herein present our experience with a case of trisomy 6 CPM that was detected through GW‐NIPT, which was associated with placental abruption. This case was approved for the publication by the Institutional Review Board at the National Center for Child Health and Development (NCCHD) (project number 2021‐056).

## 2 CASE PRESENTATION

A Japanese 42‐year‐old nulliparous woman who was conceived by *in vitro* fertilization visited our hospital at 12 weeks of gestation for consultation because NIPT conducted at 10 weeks of gestation in another clinic in Japan showed an abnormal result, specifically an increased risk of fetal trisomy 6. The test, which screened beyond aneuploidies 21, 18, 13 to all rare autosomal or sex chromosome aneuploidies—which is not common practice in Japan^4^—was positive for trisomy 6. The fetal fraction was 9.0%. The family history was unremarkable for birth defects, intellectual disability, recurrent pregnancy loss, and consanguinity. She had no abnormal prior medical, obstetrical, or gynecologic history. A first‐trimester ultrasound scan at 13+1 weeks of gestation demonstrated 2.0 mm of nuchal translucency with no structural abnormalities in the fetus and normal findings of the placenta. Amniocentesis for fetal karyotyping was completed at 16+1 weeks of gestation, and the result was 46,XX. The parent declined further testing for a uniparental disomy (UPD) 6 analysis. Ultrasonography at around 20 and 30 weeks of gestation revealed normal fetal growth without fetal malformations. The pregnancy course was uneventful until the onset of labor.

At 38+5 weeks of gestation, the pregnant woman presented to the hospital with the onset of labor pain. At the time of admission, bloody amniotic fluid was observed. Due to abnormal cardiotocography monitoring with prolonged bradycardia, an emergency cesarean section was conducted at 38+6 weeks of gestation. Placental abruption was confirmed by the observation of a blood clot attached to the placenta at delivery. Blood transfusion was not required during or after operation.

A female neonate weighing 3448 g (+2.2 SD) was delivered. The Apgar scores at 1 and 5 min were 3 and 5, respectively, and the umbilical cord arterial pH was 7.063. The infant underwent tracheal intubation immediately after birth and was admitted to the neonatal intensive care unit. The baby suffered from coagulopathy following asphyxia, which resulted in pulmonary hemorrhage, and fresh frozen plasma was administered. Mechanical ventilation was continued for 10 days. A physical examination revealed the absence of craniofacial anomalies, limb malformations, joint contractures, webbed neck, skin eruptions, and abnormal neurological findings. A brain MRI showed no findings of cerebral infarction or bleeding. Peripheral blood cell counts were normal with no blast cells or thrombocytopenia. She had hypoglycemia with high serum insulin value at 2 days of age. This was treated by the injection of glucose at a controlled concentration, and the state improved next day. She presented hypoglycemia due to transient hyperinsulinemia, but not transient neonatal diabetes mellitus. She was discharged from the hospital at 19 days of age. She showed failure to thrive at the beginning of infancy but gained weight within the normal range at 7 months of age. Her developmental achievements were normal at 10 months of age.

Prior to the delivery, the parents consented to the collection of samples of the placenta and umbilical cord blood to conduct follow‐up testing to assess the possibility of CPM and UPD6. The results of the cytogenetic analysis are summarized in Table 1. Interphase FISH studies of three placental biopsy specimens from the smooth fetal side of the placenta using CEP**^®^** 6 probes (D6Z1) specific for 6p11.1‐q11 (Abbott, Chicago, Ⅲ) revealed trisomy 6 in 3% of the total cells. The presence of only 1–5% abnormal cells in each site was not sufficient for obtain informative results from an SNP array analysis for the detection of trisomy 6 or for the determination of the origin of trisomy 6 (HumanCytoSNP‐12 DNA Analysis BeadChip Kit, Illumina). Trisomy 6 was not identified in umbilical cord or umbilical cord blood samples. These findings were consistent with CPM 6. An SNP array analysis of the umbilical cord showed the presence of a chromosome 6 pair derived from both parents; UPD6 was not detected.

**TABLE 1:** Summary of prenatal/postnatal testing results

Timing	Sample	Test	Results
Prenatal	Amniotic fluid	G‐bands	46,XX (15 cells total)
		UPD6	Declined by parents
Postnatal	Placenta	Interphase FISH	3% of trisomy 6 (10/300 cells)
		Site 1	4% of trisomy 6 (4/100 cells)
		Site 2	5% of trisomy 6 (5/100 cells)
		Site 3	1% of trisomy 6 (1/100 cells)
	Umbilical cord blood	Interphase FISH	0% of trisomy 6 (0/300 cells)
		G‐bands	46,XX (20 cells total)
	Umbilical cord	SNP array	arr(1–22,X)×2 UPD6 not detected
	Maternal peripheral blood	SNP array	arr(1–22,X)×2
	Paternal peripheral blood	SNP array	arr(1–22)×2, (X,Y)×1

Abbreviations: FISH, fluorescence in situ hybridization; SNP, single nucleotide polymorphism; UPD, uniparental disomy.

## 3 DISCUSSION

To the best of our knowledge, this is the first reported case of CPM6 detected by GW‐NIPT that was associated with placental abruption. Most pregnancies with complete trisomy 6 end with spontaneous miscarriage. If a developing fetus has mosaic trisomy 6, there is an increased chance for the pregnancy to progress and possibly survive to term.^6^ The variability in the clinical presentation is believed to be due to a trisomic chromosome of CPM or the degree of fetal mosaicism.

Placental abruption refers to the separation of the placenta from its implantation site before delivery and is associated with maternal/fetal morbidity and mortality. Several predisposing factors for it, such as prior abruption, increased age and parity, preeclampsia, and chronic hypertension, have been reported, although little is known about the association between CPM and placental abruption. Lund et al stated explicitly that there was no placental abruption in their series of cases with discordant CVS‐genetic follow‐up results.^7^ Their study included one case of trisomy 6, in which the probability of CPM was suggested, resulting in preterm birth at 26+1 weeks of gestation.^7^ In a previous reported cases involving one pregnant woman, in which NIPT versus fetal karyotyping of amniotic fluid obtained by amniocentesis revealed discordant results in one pregnant woman, suggesting the probability of CPM (trisomy 18 and XXX), an emergent delivery was required due to placental abruption.^8^ However, genetic analyses were not performed on placental samples in these two cases. We showed a case of CPM of an RAT that was associated with placental abruption. It is hypothesized that, in some cases with CPM, the presence of a trisomy in the placenta would alter some functions leading to pregnancy complications.^9^ A recent study showed that genomic alterations are often not uniformly distributed within placentas.^10^ This may explain the variable pregnancy outcomes observed in CPM. Further research is needed to quantify the risk of placental abruption in pregnant women when CPM is identified through GW‐NIPT in the clinical setting.

CPM may also be a marker for UPD, which can have clinical consequences depending on the imprinting of chromosomes 6, 7, 11, 14,15, and 20.^11^ Positive GW‐NIPT results for certain autosomal trisomies are associated with an increased risk of CPM, resulting in an increased risk for UPD. However, it is difficult to detect UPD by GW‐NIPT, as UPD does not involve an increase in the dose of the chromosome. Paternal UPD6 is associated with transient neonatal diabetes mellitus.^11^ In this case, UPD was not suggested by the blood sugar level or the results of an SNP array analysis using an umbilical cord specimen.

The present study was associated with some limitations. Although the presence of only 1–5% abnormal cells in each site was sufficient for producing an abnormal NIPT result, sampling of only 3 × 1 cm^3^ area does not exclude higher levels of trisomic cells elsewhere in the placenta. Due to the difficulty in detecting low‐level mosaicism using the microarray platform, the investigation of placental mosaicism by interphase FISH was helpful for the diagnosis of CPM, which is now recognized as the major origin of discordant NIPT results.

In conclusion, this case may imply an association between some kinds of CPM and placental abruption. It may be useful for genetic counseling of pregnant women when trisomy 6 is detected by GW‐NIPT. All pregnant women with CPM detected by NIPT should be offered additional fetal sonography to monitor the fetal growth and placental function. Larger studies are warranted to better define the associated risk of placental abruption in cases in which CPM is identified through GW‐NIPT.

## CONFLICT OF INTEREST

The authors declare no conflict of interest in association with the present study.

## AUTHOR CONTRIBUTIONS

MN involved in genetic counseling and wrote the manuscript. SW performed genetic counseling, ultrasound investigations, amniocentesis, clinical follow‐up of the patient, and wrote the manuscript. FH performed cytogenetic and molecular procedures and the analysis of the results. YU performed clinical follow‐up of the patient. MO performed cytogenetic and molecular procedures and the analysis of the results. KH supervised the cytogenetic and molecular procedures and revised the manuscript. YI followed the patient and revised the manuscript. HS performed genetic counseling, supervised the clinical management, wrote the manuscript, and finally revised the manuscript. All authors approved the final version of the manuscript.

## CONSENT

Written informed consent was obtained from the patient to publish this report in accordance with the journal's patient consent policy.

## Data Availability

The data that support the findings of this study are available from the corresponding author (SW) upon reasonable request.
